# Estimating Ground-Level Concentrations of Multiple Air Pollutants and Their Health Impacts in the Huaihe River Basin in China

**DOI:** 10.3390/ijerph16040579

**Published:** 2019-02-16

**Authors:** Deying Zhang, Kaixu Bai, Yunyun Zhou, Runhe Shi, Hongyan Ren

**Affiliations:** 1 Key Laboratory of Geographic Information Science, Ministry of Education, East China Normal University, Shanghai 200241, China; zzhangdeying@163.com (D.Z.); kxbai@geo.ecnu.edu.cn (K.B.); zzzhouyunyun@163.com (Y.Z.); 2 School of Geographic Sciences, East China Normal University, Shanghai 200241, China; 3 Joint Laboratory for Environmental Remote Sensing and Data Assimilation, East China Normal University and Institute of Remote Sensing and Digital Earth, Chinese Academy of Sciences, Shanghai 200241, China; 4 State Key Laboratory of Resources and Environmental Information System, Institute of Geographic Sciences and Natural Resources Research, Chinese Academy of Sciences, Beijing 100101, China; renhy@lreis.ac.cn

**Keywords:** geographically weighted regression, GWR, air pollution, health impacts, Huaihe River Basin

## Abstract

Air pollutants existing in the environment may have negative impacts on human health depending on their toxicity and concentrations. Remote sensing data enable researchers to map concentrations of various air pollutants over vast areas. By combining ground-level concentrations with population data, the spatial distribution of health impacts attributed to air pollutants can be acquired. This study took five highly populated and severely polluted provinces along the Huaihe River, China, as the research area. The ground-level concentrations of four major air pollutants including nitrogen dioxide (NO_2_), sulfate dioxide (SO_2_), particulate matters with diameter equal or less than 10 (PM_10_) or 2.5 micron (PM_2.5_) were estimated based on relevant remote sensing data using the geographically weighted regression (GWR) model. The health impacts of these pollutants were then assessed with the aid of co-located gridded population data. The results show that the annual average concentrations of ground-level NO_2_, SO_2_, PM_10_, and PM_2.5_ in 2016 were 31 µg/m^3^, 26 µg/m^3^, 100 µg/m^3^, and 59 µg/m^3^, respectively. In terms of the health impacts attributable to NO_2_, SO_2_, PM_10_, and PM_2.5_, there were 546, 1788, 10,595, and 8364 respiratory deaths, and 1221, 9666, 46,954, and 39,524 cardiovascular deaths, respectively. Northern Henan, west-central Shandong, southern Jiangsu, and Wuhan City in Hubei are prone to large health risks. Meanwhile, air pollutants have an overall greater impact on cardiovascular disease than respiratory disease, which is primarily attributable to the inhalable particle matters. Our findings provide a good reference to local decision makers for the implementation of further emission control strategies and possible health impacts assessment.

## 1. Introduction

Air pollutants refer to foreign substances that enter the near-surface or low-level atmospheric environment as a gaseous form. The sources can be divided into two categories: natural factors and human-induced factors. The latter is the dominant factor, and sources include industrial production, mineral combustion, automobile emissions, and so forth [1]. Many epidemiological studies have shown that air pollution in the environment has acute and chronic effects on mortality, morbidity, and hospitalization rate [2,3], and each pollutant has a certain exposure–response relationship with these health impacts [4,5]. If the PM_2.5_ concentration was reduced to the standard concentration of 10 µg/m^3^ recommended by the World Health Organization (WHO), São Paulo would avoid 5012 premature deaths each year, saving $15.1 billion [6]. However, as a country with serious air pollution and a dense population, China has more serious health problems and economic losses caused by air pollution. In 2014, 278,444 deaths and 71,058 cases of cardiovascular disease were caused by PM_2.5_ in 190 Chinese cities, and the average economic losses of amended human capital and statistical life value were 0.3% and 1% of the total GDP, respectively [7]. The majority of studies on the health impacts of air pollution in China have been concentrated on economically developed urban areas [5,8,9]. However, as an important climate transition zone, the Huaihe River Basin spans five provinces (Henan, Hubei, Anhui, Jiangsu, and Shandong) and has concentrated industry, a high population density, and serious air pollution problems. It is essential to study the environmental health impacts in this region.

Many studies have focused on the health impacts caused by one single air pollutant [10,11,12]. However, it is necessary to investigate the health impacts caused by multiple air pollutants to comprehensively understand the health risks. Regarding the air pollution data sources, many previous studies relied on observational data acquired from ambient monitoring stations to a large extent [13,14,15]. However, most of the stations in China are purposefully located in heavily polluted urban areas [16]. Therefore, the data acquired by the stations cannot well depict the spatial distribution of air pollution levels on a regional basis, and the related health impacts could be thereby overestimated if only using the monitoring station data [17,18]. Fortunately, remote sensing data can compensate for this shortcoming due to its spatial and temporal continuity, and can be applied to obtain the spatial distribution of air pollutants and the associated health impacts [19,20,21]. Satellite remote sensing products reflect the concentrations of air pollutants existing in the atmospheric column rather than at the near-surface levels which are closely related to human health. Therefore, it is necessary to further estimate the ground-level concentrations of air pollutants based on remote sensing products. The commonly used methods include numerical simulation [22,23], calculation of column quantity [21], land use regression model [24,25], geographically weighted regression (GWR) model [26,27], and geographically and temporally weighted regression (GTWR) model [28,29], etc. To assess the health impacts of air pollutants, many previous studies were mainly based on the exposure–response function of air pollutants in epidemiological studies, using risk assessment models [10,11,30] or health indicators [8,12] to obtain health risks in provinces or cities. The outputs of the assessments were usually mortality and hospitalization rate of certain diseases in a number of provinces and cities. Few studies have provided continuous spatial distribution in grids.

In this paper, five highly populated and severely polluted provinces (Henan, Hubei, Anhui, Jiangsu, and Shandong) along the Huaihe River were chosen as the research area. The ground-level concentrations of four major air pollutants including nitrogen dioxide (NO_2_), sulfate dioxide (SO_2_), particulate matters with diameter equal or less than 10 (PM_10_) or 2.5 micron (PM_2.5_) were estimated based on satellite remote sensing products and meteorological parameters in 2016. GWR was applied to obtain the ground-level concentrations of air pollutants at 0.125° grids. Then, population data were updated to 2016 and resampled to the same size. Respiratory deaths and cardiovascular deaths were two health endpoints in this study. The estimated ground-level concentrations of each air pollutant were combined with the population data to evaluate their health impacts using the health impact assessment (HIA) method. The spatial distribution characteristics of ground-level concentrations and health impacts in 2016 were analyzed. The HIA of multiple air pollutants is conducive to comparative studies and provides an in-depth understanding of the health impacts of the overall air pollution in the study area. The spatial distribution results can help to analyze regional risk differences and primary air hazards, thus helping to further facilitate targeted pollution prevention measures of the environmental protection department.

## 2. Materials and Methods

### 2.1. Study Area

**Figure 1:**
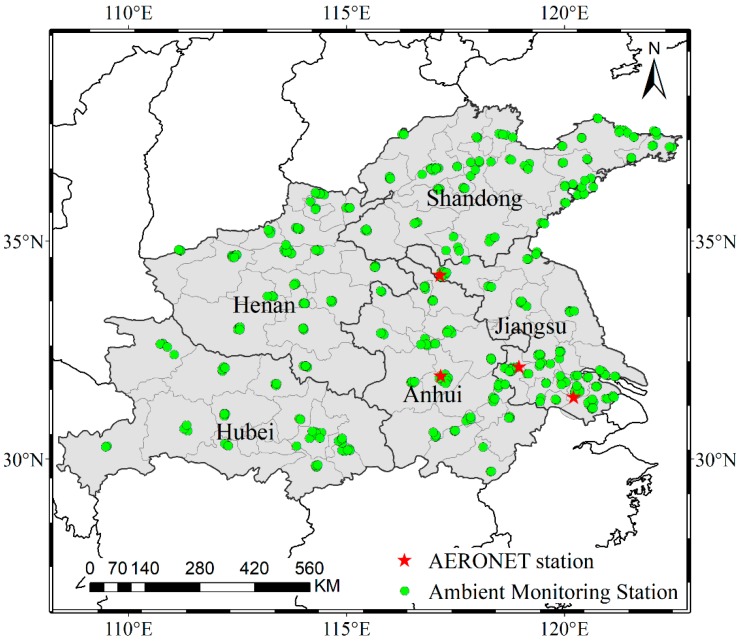
Study area and station location.

The Huaihe River Basin is located in eastern China between the Yangtze River Basin and the Yellow River Basin. The terrain varies from mountainous to hilly to plains. It is located in the north–south climate transition zone of China; north of the Huaihe River belongs to the warm temperate zone, and the south is the northern subtropical zone. The temperature rises from north to south and from the coast to the inland. The five provinces (Henan, Hubei, Anhui, Jiangsu, and Shandong) covered by the basin are the study area, with a geographic range of 29° N–38° N and 108° E–123° E (Figure 1). There are more than 70 cities in the five provinces, with a total population of more than 395 million. The average population density is 662 persons per square kilometer in the Huaihe River Basin, which is 4.8 times as many as the national average population density, and this population density is the highest among all the river basins in China [31]. The dense population, coal-based and electricity-oriented industry, and an increasing number of motor vehicles make the area one of the most severely polluted regions in China. There are 391 ambient monitoring stations located in the study area, but they are not evenly distributed.

### 2.2. Data

#### 2.2.1. Ground-Based Measurements

China established a nationwide air quality monitoring network to comprehensively assess the air quality of various regions. NO_2_, SO_2_, PM_10_, and PM_2.5_ were the main air pollutants for daily real-time monitoring. In this study, hourly measurements of NO_2_, SO_2_, PM_10_, and PM_2.5_ from 1 January 2016 to 31 December 2016 at 391 ambient monitoring stations were collected from the China National Environmental Monitoring Centre (http://www.cnemc.cn/). Due to the potential lack of measurements at certain monitoring stations in different periods, to ensure the reliability of data quality, the monitoring stations with less than 270 days of consecutive observation days were excluded. Finally, the number of effective monitoring stations for NO_2_, SO_2_, PM_10_, and PM_2.5_ was 377, 376, 379, and 379, respectively. Furthermore, the monthly average concentrations of air pollutants at the effective monitoring sites were calculated. The data were divided into two parts using random sampling: 70% were used for constructing the GWR model, and the remaining 30% were used for verifying the estimated results.

Level 1.5 ground-based aerosol optical depth (AOD) measurements for the study area were collected from the AErosol RObotic NETwork (AERONET) (http://aeronet.gsfc.nasa.gov/). These data were used to verify the reliability of the merged AOD data. However, in the study area, only four sites (Taihu, Xuzhou-CUMT, SONET_Hefei, and SONET_Nanjing) were available, and there was no corresponding AOD value for the remote sensing product at 550 nm. AERONET AOD at 550 nm was calculated by interpolating the AOD values at 440 nm and 675 nm using the provided Angstrom band conversion formula. The locations of the AERONET stations are shown in Figure 1.

#### 2.2.2. Satellite Data

- Aerosol Optical Depth

There is a certain correlation between AOD and the concentrations of ground-level particulate matters (PM_10_ and PM_2.5_). Therefore, it is common to estimate the ground-level concentrations of PM_10_ and PM_2.5_ based on the remote sensing AOD product [26,27,32]. The Moderate Resolution Imaging Spectroradiometer (MODIS) is an instrument aboard the NASA Terra and Aqua satellites, which pass over the equator at approximately 10:30 and 13:30 local time. In this study, both the daily Terra (MOD04) and Aqua (MYD04) MODIS AOD products (Collection 6.1) with 3 km spatial resolution were downloaded from the NASA LAADS website (https://ladsweb.modaps.eosdis.nasa.gov/search/order) over the study’s time period (for the year 2016). The AOD values at 550 nm were extracted from the scientific dataset “Corrected_Optical_Depth_Land” with best quality assurance and then reprojected, cropped, and spliced. Furthermore, the MOD04 and MYD04 products were merged to increase the coverage of valid data spatially and the availability temporally.

In terms of the fusion approach, pixels with valid observations co-located in MOD04 and MYDO4 were first extracted, and then an adaptive bias correction method proposed by Bai et al. (2016) [33] was applied to fuse these two AOD data sets. According to the statistics summarized in Table 1, the AOD product of MOD04 was used as the baseline data, and the AOD data of MYD04 was complementary. The reasons are as follows: (1) the MOD04 product has a larger effective pixel coverage ratio than MYD04, and (2) the AOD of MOD04 has higher accuracy (a higher correlation with ground-based AOD measurements). Specifically, seasonal common observations (i.e., co-located valid observations in both MOD04 and MYD04) were used to characterize the value-dependent bias between two data sets, and then valid observations present in MYD04 that were not observed in MOD04 were calibrated onto the MOD04 level based on common observations via quantile mapping. Finally, the corrected AOD values were merged with those of MOD04 in the spatial domain. The improved coverage ratio of valid observations and the higher correlation between merged AOD values and ground-based AOD measurements from AERONET stations fully reveal the superiority of the merged results. Finally, the results were processed to monthly mean values at a spatial resolution of 0.125° × 0.125°.

- NO_2_

The Ozone Monitoring Instrument (OMI) is an instrument onboard the NASA EOS (Earth Observation System)-Aura satellite that monitors atmospheric ozone, NO_2_, SO_2_, and other trace gases. OMI offers daily atmospheric products with global coverage at the nadir spatial resolution of 13 km × 24 km [34]. The daily Level-3 Aura OMI NO_2_ data product (OMNO2d) with 0.25° × 0.25° spatial resolution from the NASA website (http://disc.sci.gsfc.nasa.gov/datacollection/OMNO2d_003.html) contains parameters regarding the total NO_2_ columns and tropospheric NO_2_ columns. The tropospheric NO_2_ columns are retrieved based on slant columns using the differential optical absorption spectroscopy (DOAS) algorithm [35]. The fitting error in the NO_2_ slant column is estimated to be 0.3 to 1 × 10^15^ molecules/cm^2^, depending on the uncertainty of the surface albedo, NO_2_ vertical profile, and cloud interference [36,37,38]. The main sources of tropospheric nitrogen oxides include combustion, soil emissions, and lightning. Therefore, the tropospheric NO_2_ columns with screened clouds were used to estimate ground-level NO_2_ concentrations over the study’s time period (for the year 2016). The data were filtered from the Level 2 product according to the criteria that the solar zenith angle was less than 85°, the topographic reflectivity was less than 30%, and the cloud amount was less than 30% [39]. Finally, the data were processed to monthly mean values at a spatial resolution of 0.125° × 0.125°.

- SO_2_

The OMI provides SO_2_ column concentrations at four different heights: the planetary boundary layer (PBL), the bottom of the troposphere, the middle of the troposphere, and the top of the troposphere to the stratosphere. The PBL data are from heights of less than 2 km, and the center height is approximately 0.9 km. The SO_2_ within the PBL is mainly from human activities, while the SO_2_ in the higher layers is mainly from volcanic eruptions [40]. The daily Level-3 Aura OMI PBL SO_2_ data product (OMSO2e) with 0.25° × 0.25° spatial resolution from the NASA website (http://disc.sci.gsfc.nasa.gov/datacollection/OMSO2e_003.html) was applied in this study. The data are estimated from the OMSO2 L2 product using the principal component analysis (PCA) algorithm. The estimated noise standard deviation of SO_2_ is 1.2 to 1.5 DU (Dobson units, 1 DU = 2.69 × 10^16^ molecules/cm^2^); this noise can be decreased to less than 0.3 DU after averaging the data in space and time [41,42]. The quality of SO_2_ satellite data was generally poor, and there were a large number of missing values and noisy points. Therefore, preprocessing and quality control were required to eliminate poor-quality pixels in this study. The screening criteria were as follows: (1) radiative cloud fraction less than 0.2, and (2) solar zenith angle less than 70 degrees. Furthermore, spatial interpolation and spatial filtering were used to improve the range and quality of the available data. The PBL SO_2_ columns excluding missing data and low-quality data were used to estimate ground-level SO_2_ concentrations in 2016. The data were processed to monthly mean values at a spatial resolution of 0.125° × 0.125°.

#### 2.2.3. Meteorological Data

Because the accumulation, diffusion, and evolution of air pollutants are highly related to weather conditions, it is necessary to consider the effects of various meteorological factors when estimating the ground-level concentrations of air pollutants based on remote sensing products. We selected temperature (T), relative humidity (RH), U-wind speed (U), V-wind speed (V), and boundary layer height (BLH) as the impact factors based on a comprehensive review of previous studies [23,29,43]. These meteorological parameters were collected from the European Centre for Medium-Range Weather Forecasts (ECMWF) ERA-Interim reanalysis with a spatial resolution of 0.125° × 0.125°. All of the meteorological data were monthly means of daily means. They were available from the ECMWF website (http://apps.ecmwf.int/datasets/data/interim-full-daily).

#### 2.2.4. Population Data

**Figure 2:**
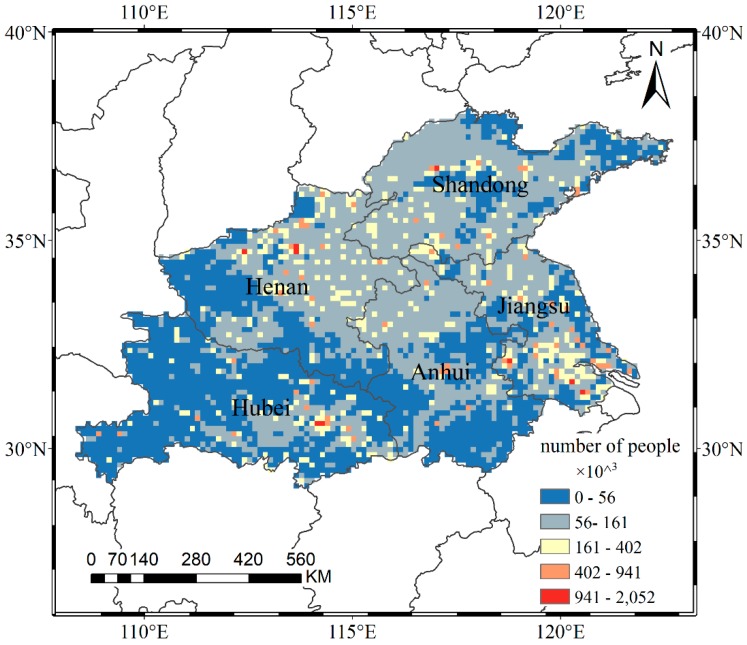
Spatial distribution of the population in 2016.

The population grid data with a 1 km resolution in 2010 was provided by the Resource and Environmental Science Data Center of the Chinese Academy of Sciences (RESDC) (http://www.resdc.cn/). Based on the demographic data at the county level, the dataset uses the multifactor weight distribution method to assign the population in administrative units to 1 kilometer grids, taking into account the land use type, nighttime lighting, and residential density [44]. We converted the grid to 0.125° to match the spatial resolution of the air pollutant data mentioned above. We updated the population data to 2016 based on the annual provincial population data released by the Chinese National Bureau of Statistics (http://www.stats.gov.cn/tjsj/ndsj/). The result is shown in Figure 2. The result was used in conjunction with estimated ground-level concentrations of air pollutants to assess the health impacts in exposed populations. The main population update steps were as follows: (1) the regional statistical method was used to obtain the population distribution in 2010 with a resolution of 0.125°; (2) the proportion of each grid population to the total population of the corresponding province in 2010 was calculated, labeled A; (3) the proportion A was multiplied by the total population B of the corresponding province in 2016, and then the population grid data was obtained at 0.125° for the study area in 2016.

### 2.3. Methods

#### 2.3.1. Geographically Weighted Regression

GWR is a practical technique to detect the spatial variability and non-stationarity of continuous surfaces of regional parameters by generating local regression results [45,46]. Compared with the traditional linear and nonlinear regression models, the GWR determines the spatial variation in the regression model coefficients, which significantly improves the estimation accuracy of the model. GWR was applied in the estimation of ground-level concentrations of air pollutants in this study. As we know, remote sensing products reflect the column concentration of the air pollutants within a certain height in the atmosphere. However, human health is mostly related to the ground-level concentration of air pollutants. Therefore, it is necessary to estimate the ground-level concentrations of air pollutants based on remote sensing products. Some previous studies have shown that the correlations between AOD and PM_2.5_ and PM_10_ varied distinctively in different places [47]. The spatial variation in correlation may lead to inaccurate estimation of the model when using a global parameter [26,43]. The correlation between AOD and PM_2.5_ should not be constant across space, but should change with the spatial environment [43]. GWR takes into account the local effect of spatial objects, and uses the relevant information of adjacent regions to estimate local regression parameters, and finally realizes that the coefficients of the regression model in different regions change with the change of spatial location [48]. GWR is widely used to predict the spatial distribution of pollutants concentration [49,50]. Therefore, this study used the GWR model to estimate the ground-level concentrations of PM_2.5_, PM_10_, NO_2_, and SO_2_. Both the satellite data and meteorological parameters were entered into the GWR model. GWR was used to generate a local regression coefficient for each ambient monitoring station on a monthly basis. The structure of the GWR model for multiple air pollutants in this study can be expressed as Equations (1) to (4):

(1) $$PM_{2.5,i,m}=\alpha_{0,i,m}+\alpha_{1,i,m}AOD_{i,m}+\alpha_{2,i,m}RH_{i,m}+\alpha_{3,i,m}T_{i,m}+\alpha_{4,i,m}U_{i,m}+\alpha_{5,i,m}V_{i,m}+\alpha_{6,i,m}BLH_{i,m}$$

(2) $$PM_{10,i,m}=\beta_{0,i,m}+\beta_{1,i,m}AOD_{i,m}+\beta_{2,i,m}RH_{i,m}+\beta_{3,i,m}T_{i,m}+\beta_{4,i,m}U_{i,m}+\beta_{5,i,m}V_{i,m}+\beta_{6,i,m}BLH_{i,m}$$

(3) $$NO_{2_{\_ground},i,m}=b_{0,i,m}+b_{1,i,m}NO_{2_{\_trop},i,m}+b_{2,i,m}RH_{i,m}+b_{3,i,m}T_{i,m}+b_{4,i,m}U_{i,m}+b_{5,i,m}V_{i,m}+b_{6,i,m}BLH_{i,m}$$

(4) $$SO_{2_{\_ground},i,m}=p_{0,i,m}+p_{1,i.m}SO_{2{\_}_{PBL},i,m}+p_{2,i,m}RH_{i,m}+p_{3,i,m}T_{i,m}+p_{4,i,m}U_{i,m}+p_{5,i,m}V_{i,m}+p_{6,i,m}BLH_{i,m}$$

where $PM_{2.5,i,m}$ (µg/m^3^), $PM_{10,i,m}$ (µg/m^3^), $NO_{2_{\_ground},i,m}$ (µg/m^3^), and $SO_{2{\_}_{ground},i,m}$ (µg/m^3^) are the monthly ground-based PM_2.5_, PM_10_, NO_2_, and SO_2_ concentrations at location *i* in month m, respectively; $\alpha_{0,i,m}$, $\beta_{0,i,m}$, $b_{0,i,m},$ and $p_{0,i,m}$ denote the intercepts at location *i* in month m of PM_2.5_, PM_10_, NO_2_, and SO_2_, respectively; $\alpha_{1,i,m}$ to $\alpha_{6,i,m}$, $\beta_{1,i,m}$ to $\beta_{6,i,m}$, $b_{1,i,m}$ to $b_{6,i,m}$, and $p_{1,i,m}$ to $p_{6,i,m}$ represent location-specific slopes of corresponding parameters in month m; $AOD_{i,m}$ (no unit) refers to the merged AOD from Terra and Aqua at location *i* in month m; $NO_{2_{\_trop},i,m}$ (molecules/cm^2^) refers to the OMI tropospheric NO_2_ columns at location *i* in month m; $SO_{2{\_}_{PBL},i,m}$ (DU) refers to the OMI PBL SO_2_ columns at location *i* in month m; $RH_{i,m}$ (%), $T_{i,m}$ (K), $U_{i,m}$ (m/s), $V_{i,m}$ (m/s), and $BLH_{i,m}$ (m) represent the following meteorological parameters: relative humidity, temperature, U-wind speed, V-wind speed, and boundary layer height at location *i* in month m, respectively.

#### 2.3.2. Health Impact Assessment

To quantitatively account for the human health impacts of exposure to multiple air pollutants, the HIA method was used in this study based on the concentrations of air pollutants, exposure–response functions, and gridded population data. This method has been widely used in previous assessments of air pollution hazards [51,52,53]. The preconditions of the HIA method are as follows in this study: (1) the exposure–response coefficient is the value of long-term exposure, the annual average concentration of air pollutants used in calculating the relative risk; (2) the health impact assessment unit can be changed from the province or city to the grid; (3) if the air pollutant concentration exceeds the safe threshold, the health risk of the total population in the grid area can be calculated based on the risk in 1 µg/m^3^ concentration increments for one person in epidemiological studies.

Two health endpoints were selected in this study to assess the health impacts caused by PM_2.5_, PM_10_, NO_2_, and SO_2_: respiratory deaths and cardiovascular deaths.

First, the relative risk (RR) of a certain pollutant for the health outcomes can be calculated as follows:

(5) $$\mathrm{RR}=\exp\left[\beta \times \left(C-C_{0}\right)\right]$$

where C represents the annual average concentration of an air pollutant; $C_{0}$ is the reference safe threshold concentration of health hazards resulting from the pollutant, with its value based on the air quality guidelines of the WHO [54]; β is the exposure–response coefficient, which is the percentage increase in health impacts per 1 µg/m^3^ specific air pollutant concentration increment. The β value is based on the meta-analysis results of the latest relevant literature on residents in China [55]. The literature retrieved the epidemiological studies published at home and abroad concerning the impact of air pollutants on mortality from cardiovascular disease and respiratory disease in the past decade, and screened 23 papers with the final data. The percentage increase in disease mortality per unit concentration of air pollutants in each paper and its 95% CI were used. Reference data were quantitatively synthesized through a meta-analysis to obtain the final exposure–response relationship between air pollution and human mortality impacts in China. The coefficient results were compared with the meta-analysis results of the relevant literature at home and abroad [56,57]. The gap is within a reasonable range, and the coefficient results have been cited by other literature [58], ensuring the applicability and validity of the data. The β values and $C_{0}$ values of PM_2.5_, PM_10_, NO_2_, and SO_2_ are listed in Table 2.

The number of cases E for each health endpoint attributed to a specific air pollutant is calculated using Equation (6):

(6) $$E=P\times \left(f_{p}-f_{0}\right)$$

where P is the exposed population; $f_{p}$ represents the current baseline incidence, which can be calculated by multiplying the incidence rate in a clean environment $f_{0}$ by the RR:

(7) $$f_{p}=f_{0}\times \mathrm{RR}$$

Finally, by combining Equations (6) and (7), we can calculate E as formula (8):

(8) $$E=f_{p}\times P\times \left(\frac{RR-1}{RR}\right)$$

The current baseline incidence $f_{p}$ of specific diseases can be obtained from the China Health and Family Planning Statistics Yearbook.

## 3. Results

### 3.1. GWR Model Results and Verification

The NO_2_, SO_2_, and AOD values retrieved from satellite data reflect the air pollution status from the land surface to the top of the atmosphere. However, people only breath the air at ground level. Therefore, the GWR model is applied to convert column concentrations into ground-level concentrations of air pollutants. In this study, 70% of the ambient monitoring station data were used for GWR modeling, and the remaining 30% were used for validation. Three statistical evaluation indicators, the coefficient of determination (*R*^2^), the root mean square error (RMSE), and the mean absolute percentage error (MAPE), were calculated to compare the estimated results and measured values.

**Figure 3:**
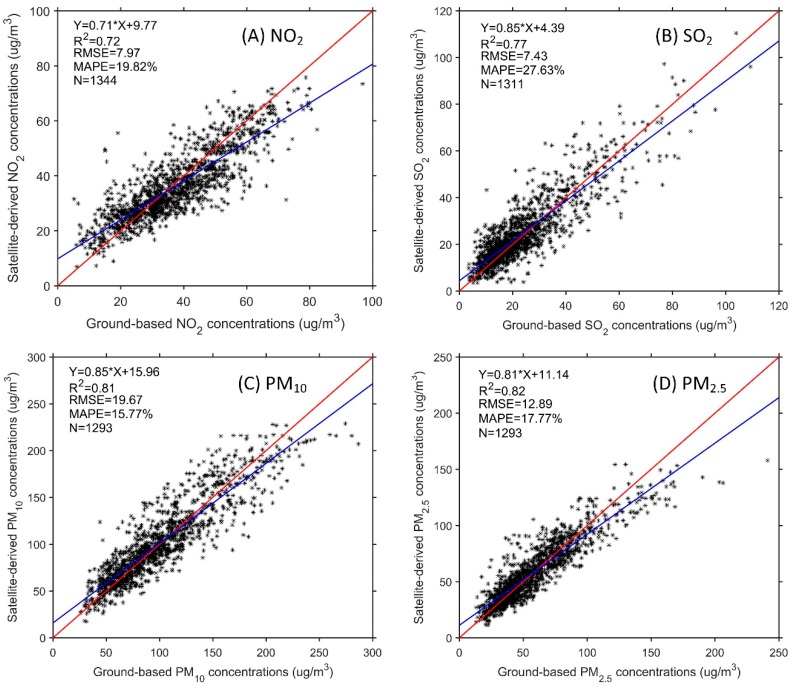
Comparison of satellite-derived air pollutant concentrations (**A**) NO_2_, (**B**) SO_2_, (**C**) PM_10_, (**D**) PM_2.5_ with ground-based concentrations.

The modeling and verification results of the GWR model are listed in Table 3. All the *R*^2^ are higher than 0.70, which means that the conversion results were satisfactory, and the *R*^2^ values for PM_10_ and PM_2.5_ are slightly higher than those for NO_2_ and SO_2_. Scattering plots for verification are depicted in Figure 3. The blue line (linear fitting) is close to the red line (1:1), indicating that the results are reasonable. The value of three statistical evaluation indicators for verification show that there is a high correlation and low deviation between the estimated results and measurements. Therefore, the estimated ground-level concentrations of air pollutants is close to the measurements, ensuring the applicability and reliability of the data sources used for health impact assessment.

### 3.2. Spatial Distribution of Ground-Level Air Pollutants

#### 3.2.1. Spatial Distribution of Ground-Level PM_10_ and PM_2.5_

**Figure 4:**
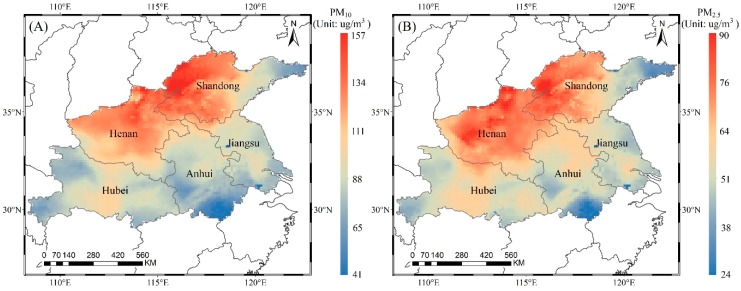
Spatial distribution of ground-level PM_10_ (**A**) and PM_2.5_ (**B**) in 2016.

As major forms of inhalable particulate matter, PM_10_ and PM_2.5_ are mainly derived from human activities, such as coal combustion, industrial production, and vehicle emissions. They affect human health via deposition in the respiratory tract. Based on equations (1) and (2), we estimated the ground-level PM_2.5_ and PM_10_ concentrations, respectively. The spatial distribution of the annual average concentration in 2016 is shown in Figure 4 with a spatial resolution of 0.125° × 0.125°. The annual average concentrations of PM_10_ and PM_2.5_ in the study area are 100 µg/m^3^ and 59 µg/m^3^, respectively. Because PM_10_ and PM_2.5_ are both estimated based on AOD products, there is a similarity in the spatial distribution trends, but the numerical difference is very large. The highest annual average concentrations of PM_10_ and PM_2.5_ are up to 157 µg/m^3^ and 90 µg/m^3^, respectively. The percentage of the total study area with PM_10_ concentrations exceeding the Chinese National Secondary Air Quality Standard (CNSAQS, GB3095-2012) of 70 µg/m^3^ is approximately 93%. The remaining areas are mainly coastal areas (Weihai city in eastern Shandong) and mountains (Huangshan city in southern Anhui) due to the mitigation of sea breezes and forests. The most highly polluted areas of PM_10_ are concentrated in Henan and west-central Shandong, where the concentrations exceed 120 µg/m^3^. They are also the most polluted areas for PM_2.5_, with concentrations twice as high as those of CNSAQS (35 µg/m^3^). Approximately 98% of the entire study area has PM_2.5_ concentrations exceeding the CNSAQS, which is greater than that for PM_10_. In general, in terms of inhalable particulate matter, the five provinces along the Huaihe River are seriously polluted. These conditions are mainly related to serious industrial and agricultural pollution in the region, which may cause serious harm to the environment and human health in the study area. It is necessary to control and prevent heavy pollution in these areas.

#### 3.2.2. Spatial Distribution of Ground-Level NO_2_ and SO_2_

**Figure 5:**
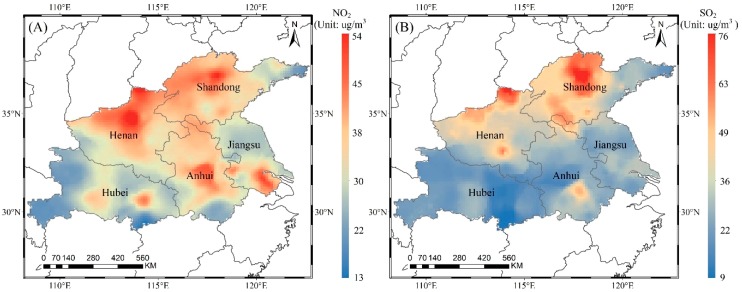
Spatial distribution of ground-level NO_2_ (**A**) and SO_2_ (**B**) in 2016.

NO_2_ and SO_2_ are important pollution gases in the troposphere and are mainly from automobile emissions and fossil fuel combustion. They can be oxidized to form sulfate aerosols and nitrate aerosols, which constitute the main components of haze. The tropospheric NO_2_ and PBL SO_2_ were estimated to the ground level based on equations (3) and (4), respectively. Figure 5 reveals the spatial distribution of the annual average concentrations of NO_2_ and SO_2_ in 2016, with a spatial resolution of 0.125° × 0.125°. The annual average concentrations of NO_2_ and SO_2_ in the study area are 31 µg/m^3^ and 26 µg/m^3^, respectively. The highest annual average concentrations of NO_2_ and SO_2_ are up to 54 µg/m^3^ and 76 µg/m^3^, respectively. The NO_2_ concentrations in the study area exceed the CNSAQS (40 µg/m^3^) by approximately 15%, especially in the large and medium-sized cities in various provinces, such as Zhengzhou in Henan, Zibo in Shandong, and Hefei in Anhui. The pollution levels are due to the dense population and industrial development in these cities. In space, the concentration of NO_2_ gradually decreases from cities with higher concentrations to the surrounding areas with lower concentrations. The spatial distribution characteristics are mainly because the lifetime of NO_2_ in the air is on the order of hours, so the highly concentrated areas are mainly located around the emission sources.

The annual average concentration of SO_2_ varies widely between regions, with concentrations ranging from 9 µg/m^3^ to 76 µg/m^3^. The high-value areas are mainly located in north-central Shandong, where the SO_2_ concentration exceeds the CNSAQS (60 µg/m^3^). These high pollution levels are related to the heavy industrial production in the region. In Hubei, Anhui, Jiangsu, and eastern Shandong, the SO_2_ concentrations are below 35 µg/m^3^. The area in the five provinces along the Huaihe River with SO_2_ concentrations exceeding the CNSAQS is less than 1%. Therefore, there is basically no SO_2_ pollution. In general, the NO_2_ and SO_2_ pollution in the five provinces along the Huaihe River is lighter than the PM_10_ and PM_2.5_ pollution.

### 3.3. Health Impact Assessment

**Figure 6:**
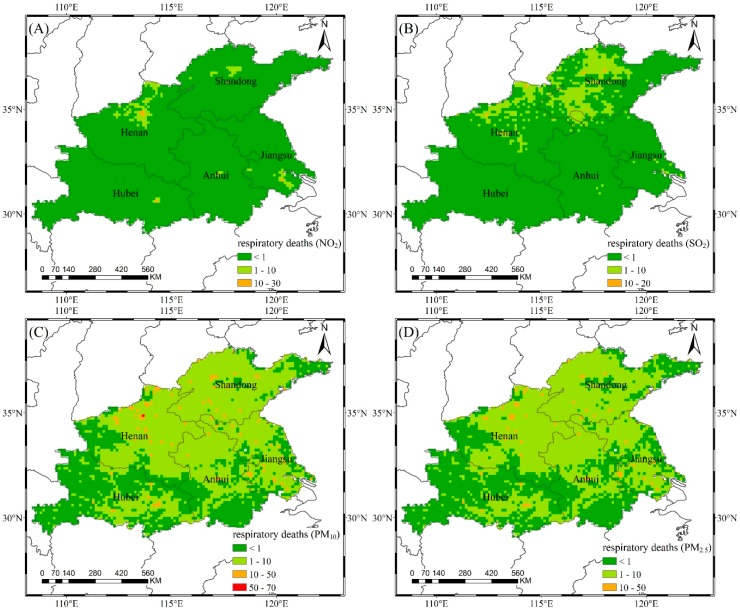
The number of respiratory deaths attributed to NO_2_ (**A**), SO_2_ (**B**), PM_10_ (**C**), and PM_2.5_ (**D**).

**Figure 7:**
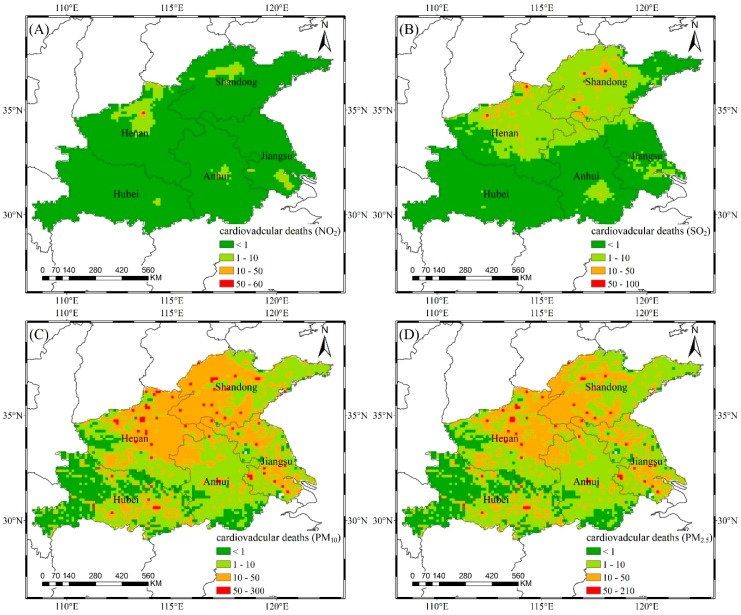
The number of cardiovascular deaths attributed to NO_2_ (**A**), SO_2_ (**B**), PM_10_ (**C**), and PM_2.5_ (**D**).

Air pollutants carry toxic substances into the human body through chemical reactions, causing physiological dysfunction in the human respiratory system or other systems. Compared with other pollutants, PM_2.5_ (as a form of inhalable particulate matter) has a small particle size, high adhesiveness, and strong penetrating power. PM_2.5_ can even enter the circulatory system, where it can bring about serious cardiovascular diseases, respiratory diseases, and even lung cancer. Based on Equation (8), we combined the concentrations of air pollutants with population exposure information and then obtained the number of cases for two health endpoints associated with air pollutants in the study area, as shown in Figure 6 and Figure 7, respectively. The spatial resolution is 0.125° × 0.125°. The value indicates the increased number of disease deaths due to current air pollutant concentrations using the air quality guidelines of WHO as reference concentrations. Table 4 shows the total number of disease deaths caused by air pollutants in 2016.

In terms of the respiratory deaths associated with air pollutants, PM_10_ has the largest impact value and range. Shandong and Henan are the most affected regions, with 3139 and 3147 respiratory deaths attributable to PM_10_, respectively, accounting for approximately 0.4% of the total death tolls in the corresponding provinces. In terms of spatial distribution, the high-value areas are mainly represented by a number of discrete pixels because of the concentrated population distribution (as shown in Figure 2). Wuhan in Hubei has less than one respiratory death attributed to SO_2_ because the concentrations are less than 35 µg/m^3^, even with the unit pixel population of 2.05 million. The concentrations of air pollutants in west-central Shandong exceeds the CNSAQS, but southern Jinan and southern Zibo each only have one respiratory death, because the unit pixel population are less than 50,000. The health impacts are the combined result of air pollutant concentration and population density. Therefore, it is necessary to take targeted measures in the region, and the relevant actors should not rely solely on population distribution or pollution levels.

Air pollution produces more cardiovascular deaths than respiratory deaths. The affected area is wide, extending from eastern Henan to western Shandong, with 10 to 50 cardiovascular deaths attributable to PM_10_ and PM_2.5_. The highest pixel values for PM_10_ and PM_2.5_ are 280 and 206, respectively. The number of cardiovascular deaths caused by PM_10_ in Shandong and Henan account for 1.9% and 1.8% of the total death tolls, respectively. Although the contaminated area of SO_2_ is smaller than that of NO_2_, the health risk of SO_2_ is greater than that of NO_2_ when combined with the population distribution. The high-risk area of SO_2_ covers northern Henan and central Shandong. Zhengzhou in Henan has the highest risk attributable to PM_10_ and PM_2.5_, with 1436 and 1135 cardiovascular deaths, respectively. These high values are due to the high PM_10_ and PM_2.5_ concentrations and the large population. The risk value of cardiovascular disease is also related to the pollutant concentrations and the population distribution. Southern Anhui and western Hubei are mostly mountainous and hilly, with sparse populations and low air pollutant concentrations. Therefore, the health risks attributed to air pollution are also low.

In general, the health impacts of air pollution are mainly concentrated in northern Henan, west-central Shandong, southern Jiangsu, and Wuhan in Hubei. The health impacts of air pollutants are mainly related to inhalable particulate matter. The main reason is that the concentrations of PM_10_ and PM_2.5_ in the study area are too high. If the air quality guidance value of the WHO is the reference concentration, the annual average concentrations of PM_10_ and PM_2.5_ exceed the reference value by 5 times and 6 times, respectively, while the NO_2_ and SO_2_ concentrations are close to the reference concentrations. In addition, the properties of inhalable particulate matter also represent another reason behind the health impacts. PM_2.5_ has a large surface area onto which various pollutants can be adsorbed to form a synergistic effect of multiple pollutants. The heart and blood vessels are vulnerable to air pollution, and the resulting mortality is high. The degree and scope of health effects attributed to air pollution in different regions vary, and appropriate preventive measures should be taken in a targeted manner.

## 4. Discussion

Our research provided spatially continuous information on the health impacts associated with four major air pollutants in the five provinces along the Huaihe River. However, there are some limitations. First, instead of a physical model, a geographic statistical model was applied to estimate the ground-level concentrations of air pollutants. Considering the complicated interactions between air pollutants and meteorological parameters, it is difficult to depict the change and trajectories of various air pollutants effectively and efficiently in our research area for the whole year. Moreover, the *R*^2^ value between the estimated concentration of air pollutants and the measured value for the ground sites (Table 3) is high enough to prove that the empirical model is credible.

Second, the exposure–response coefficients we used are referenced from previous literature, and the actual coefficients in the study area may differ due to the differences in individual populations, regional environments, and living habits. At present, there are few studies on the exposure–response coefficients of air pollution and population health effects in various regions in China. However, the data are from published meta-analysis results in China. Use of these data could reduce the uncertainty of the coefficients in the region. Our health impact assessment results are consistent with previous studies. For example, Fang et al. (2016) [14] found that PM_2.5_ caused more than 3000 cardiovascular deaths and 150 respiratory deaths in Suzhou, Jinan, and Hefei by analyzing the health effects attributed to PM_2.5_ in 74 major cities in China in 2013. Cardiovascular disease contributed the most to the total PM_2.5_-related deaths, and large numbers of disease cases were mostly found in developed metropolitan regions. In addition, Song et al. (2016) [59] reported that there were approximately 1000 cases of cardiovascular disease and 150 cases of respiratory disease caused by PM_2.5_ in Wuhan. In our research, in Suzhou, Jinan, and Wuhan, the number of respiratory diseases attributed to PM_2.5_ was 230, 139, and 158, respectively, and the number of cardiovascular diseases was 1087, 656, and 750, respectively. Furthermore, we evaluated the health effects of multiple air pollutants and analyzed their spatial distribution differences.

There is a certain correlation between air pollutants. Specifically, there is a strong correlation between PM_2.5_ and PM_10_ (the correlation coefficient is greater than 0.8). The annual average concentrations of PM_2.5_ and PM_10_ are also positively correlated with NO_2_ and SO_2_ [60]. This is related to common sources of contamination and photochemical reaction between air pollutants [61]. Our research achieved a single health impact of multiple air pollutants, rather than a comprehensive health impact. The interaction of other contaminants was ignored in conducting the health impact assessment of one air pollutant. In theory, there is a risk of double counting. The overall health risks related to air pollution in the study area may be lower than the evaluation results. The results of this study will be mainly useful for roughly estimating the health impacts caused by air pollutants, generally clarifying the spatial distribution, and serving as reference values for the decision-making of relevant departments. The interaction of air pollutants and the impact of integrated pollution on human health will be the foci of future research studies.

## 5. Conclusions

Based on satellite observation data and various meteorological parameters, this study used a GWR model to estimate the ground-level concentrations of NO_2_, SO_2_, PM_10_, and PM_2.5_ in five provinces along the Huaihe River in 2016. Furthermore, these pollution data were combined with population data to analyze the health impacts of exposure to various air pollutants, and respiratory deaths and cardiovascular deaths were chosen as two health endpoints. The results show that the ground-level concentrations of air pollutants can be reliably obtained based on the relevant satellite observation data. The estimation results can explain more than 72% of the station measurements of each air pollutant. The annual average concentrations of NO_2_, SO_2_, PM_10_, and PM_2.5_ in the study area are 31 µg/m^3^, 26 µg/m^3^, 100 µg/m^3^, and 59 µg/m^3^, respectively. The areas with PM_10_ and PM_2.5_ concentrations exceeding the CNSAQS both exceeded 90%.

From the results of related health impacts, NO_2_, SO_2_, PM_10_, and PM_2.5_ are associated with 546, 1788, 10,595 and 8364 respiratory deaths, and 1221, 9666, 46,954 and 39,524 cardiovascular deaths, respectively. These air pollutants are more likely to have greater impacts on cardiovascular disease than respiratory disease. The health impacts of air pollution are mainly concentrated in northern Henan, west-central Shandong, southern Jiangsu, and Wuhan in Hubei, where the health impacts for individual cities are particularly evident. Heavily impacted cities mainly include Zhengzhou, Luoyang, Jinan, Jining, Nanjing, Wuxi, and Wuhan, which have dense populations and high concentrations of air pollutants. In contrast, southern Anhui and western Hubei are mostly mountainous and hilly, where health risks attributed to air pollution are low. The health impacts are the combined results of air pollutant concentrations and population density.

The areas affected by PM_10_ and PM_2.5_ cover almost all of the five provinces along the Huaihe River. Therefore, the five provinces along the Huaihe River should consider PM_10_ and PM_2.5_ as the priority pollutants to be controlled. The health impacts caused by NO_2_ and SO_2_ are relatively low, indicating the effectiveness of air pollution control implemented in the study area. However, due to the problems of national energy and industrial structure, the governance of particulate matter pollution may take a longer time. Pollution control in higher health hazard areas should be given more attention.

## Figures and Tables

**Table 1:** Statistics of satellite-based aerosol optical depth (AOD) and merged AOD products.

AOD	Days with Valid Observations	Effective Pixel Coverage	Sample Size, *N*	Correlation Coefficient, *R*
MYDO4	366	23.93%	133	0.81
MOD04	357	26.22%	147	0.79
Merged	366	33.46%	196	0.83

**Table 2:** Reference threshold and exposure–response coefficients of the multiple air pollutants.

Air Pollutants	$C_{0}$ (µg/m^3^)	β (%) for Respiratory Disease (95% Confidence Interval)	β (%) for Cardiovascular Disease (95% Confidence Interval)
PM_2.5_	10	0.056 (0.039, 0.081)	0.075 (0.045, 0.125)
PM_10_	20	0.043 (0.023, 0.080)	0.054 (0.032, 0.091)
NO_2_	40	0.183 (0.108, 0.310)	0.115 (0.083, 0.161)
SO_2_	20	0.083 (0.021, 0.322)	0.127 (0.093, 0.172)

$C_{0}:$ the safe threshold concentration; β: the exposure-response coefficient.

**Table 3:** Model fitting and verification of air pollutants estimation.

Air Pollutants	Modeling		Verification	
	*N*	*R* ^2^	*N*	*R* ^2^
NO_2_	3136	0.75	1344	0.72
SO_2_	3067	0.79	1311	0.77
PM_10_	3016	0.84	1293	0.81
PM_2.5_	3016	0.83	1293	0.82

**Table 4:** Increased number of disease deaths caused by exposure to air pollutants.

Air Pollutants	Increased Respiratory Deaths	Increased Cardiovascular Deaths
NO_2_	546	1221
SO_2_	1788	9666
PM_10_	10,595	46,954
PM_2.5_	8364	39,524
